# mTOR Cross-Talk in Cancer and Potential for Combination Therapy

**DOI:** 10.3390/cancers10010023

**Published:** 2018-01-19

**Authors:** Fabiana Conciatori, Ludovica Ciuffreda, Chiara Bazzichetto, Italia Falcone, Sara Pilotto, Emilio Bria, Francesco Cognetti, Michele Milella

**Affiliations:** 1 Medical Oncology 1, IRCCS Regina Elena National Cancer Institute, Rome 00144, Italy; fabiana.conciatori@ifo.gov.it (F.C.); chiara.bazzichetto@ifo.gov.it (C.B.); italia.falcone@ifo.gov.it (I.F.); francesco.cognetti@ifo.gov.it (F.C.); michele.milella@ifo.gov.it (M.M.); 2 Department of Medical-surgical Sciences and Translational Medicine, University of Rome, La Sapienza, Rome 00185, Italy; 3 Department of Molecular Medicine, University of Rome, La Sapienza, Rome 00185, Italy; 4 Medical OncologyUnit, Azienda Ospedaliera Universitaria Integrata, University of Verona, Verona 37100, Italy; sara.pilotto.85@gmail.com (S.P.); emiliobria@yahoo.it (E.B.)

**Keywords:** mTORC1, mTORC2, cancer, cross-talk, targeted therapies

## Abstract

The mammalian Target of Rapamycin (mTOR) pathway plays an essential role in sensing and integrating a variety of exogenous cues to regulate cellular growth and metabolism, in both physiological and pathological conditions. mTOR functions through two functionally and structurally distinct multi-component complexes, mTORC1 and mTORC2, which interact with each other and with several elements of other signaling pathways. In the past few years, many new insights into mTOR function and regulation have been gained and extensive genetic and pharmacological studies in mice have enhanced our understanding of how mTOR dysfunction contributes to several diseases, including cancer. Single-agent mTOR targeting, mostly using rapalogs, has so far met limited clinical success; however, due to the extensive cross-talk between mTOR and other pathways, combined approaches are the most promising avenues to improve clinical efficacy of available therapeutics and overcome drug resistance. This review provides a brief and up-to-date narrative on the regulation of mTOR function, the relative contributions of mTORC1 and mTORC2 complexes to cancer development and progression, and prospects for mTOR inhibition as a therapeutic strategy.

## 1. Introduction

mTOR is a serine/threonine kinase with high evolutionary conservation from yeast to humans with a crucial role in the integration of intracellular/extracellular growth signals and cellular metabolism [1,2]. mTOR acts through two structurally and functionally distinct enzyme complexes (mTORC1 and mTORC2) regulating protein synthesis, cell growth, metabolism, homeostasis, survival, autophagy and response to stress [3]. Nevertheless, it remains to be clarified whether mTOR has a complex-independent role in regulating cell behaviors. 

**Figure 1:**
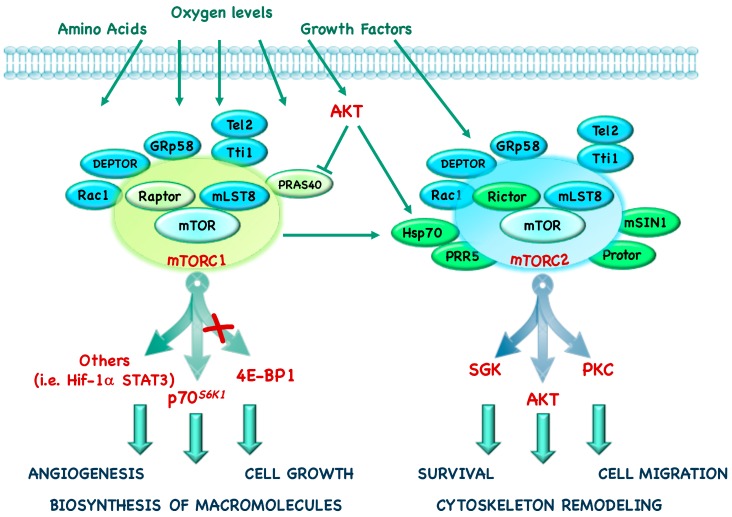
mammalian Target of Rapamycin (mTOR) complexes. mTOR protein forms two unique complexes, called mTOR complex (mTORC)1 and mTORC2. mTORC1 is activated by growth factors, amino acids, and energy levels, whereas mTORC2 is primarily responsive to growth factors. mTORC1 is comprised of the core proteins mTOR, Regulatory-associated protein of mTOR (Raptor), and mLST8; mTORC1 also binds other proteins in a species- and condition-specific manner: Proline-Rich AKT Substrate 40 (PRAS40), DEP domain-containing mTOR interacting protein (DEPTOR), GRp58, Tel2-interacting protein 1 (Tti1)- Telomere maintenance 2 (Tel2) and Rac1. mTORC2 includes the core proteins mTOR, Rapamycin insensitive companion of mTOR (Rictor), and mammalian Lethal with Sec13 protein 8 (mLST8), as well as various associated proteins, Proline-Rich Protein (PRR)5, Heat shock protein (Hsp) 70, DEPTOR, GRp58, Tti1-Tel2, Rac1, mammalian Stress-activated protein kinase Interacting protein (mSIN) 1 and Protein observed with RICTOR (Protor). mTORC1 regulates numerous processes including cell growth and proliferation, biosynthesis of macromolecules (proteins, DNA, and lipid synthesis), and angiogenesis, by regulating p70 ribosomal protein S6 Kinase 1 (p70*^S6K1^*) and Elongation Initiation Factor (EIF)-4E Binding Protein 1 (4E-BP1). mTORC2 controls cell structure, cytoskeletal reorganization, and survival by activating Serum and Glucocorticoid Kinase (SGK), Protein kinase B (AKT), and Protein-Kinase C (PKC).

mTORC1 is defined by the presence of its core component: mTOR, the scaffolding protein Regulatory-associated protein of mTOR (Raptor) and the mammalian Lethal with Sec13 protein 8 (mLST8, also known as GβL) [4,5,6]. In addition to these components, mTORC1 also contains Telomere maintenance 2 (Tel2), Tel2-interacting protein 1 (Tti1), Rac1, GRp58, and two negative regulators, DEP domain-containing mTOR interacting protein (DEPTOR) and Proline-Rich Protein kinase B (AKT) Substrate 40 (PRAS40) (Figure 1) [7,8,9,10,11,12].

The mTORC2 core complex contains mTOR, Rapamycin insensitive companion of mTOR (Rictor), mLST8 and the mammalian Stress-activated protein kinase Interacting protein 1 (mSIN1) [13]. In addition, mTORC2 also contains the specific proteins Heat shock protein (Hsp) 70, Protein observed with RICTOR (Protor) 1/2, Proline-Rich Protein (PRR) 5 and the common proteins Tti1, Tel2, DEPTOR (Figure 1) [14,15,16]. 

mTORC1 and mTORC2 have different physiological functions, they are activated in different manners and have distinct substrate specificity, therefore, their functions are strictly regulated. The mTOR signaling pathway is a nutrient switch regulated by growth factors, amino acids, bioenergetic signals and oxygen levels. Signaling through mTOR modulates a wide range of cell growth-related processes. Basically, there are different important pathways that regulate mTOR activity including the phosphoInositide3-Kinase (PI3K)/Protein kinase B (AKT), Tuberous Sclerosis Complexes (TSC) 1/2, pathway and DEPTOR loop [2,17,18].

Deregulation of the mTOR pathway is associated with several metabolic and degenerative human diseases, including cancer; moreover, recent evidence shows that both high (i.e., cancer and neurological diseases) and low (i.e., atrophy of the muscle in certain situations and aging) levels of mTOR activity are implicated in different physiological and pathological processes. However, how these changes in mTOR activity are involved in the pathogenic mechanisms of these diseases is less clear: presumably, the different effects of mTOR activity are correlated with processes downstream of mTOR which are likely interconnected to all these diseases [2].

Given the many oncogenes or tumor suppressors linked to mTOR signaling, it is estimated that mTORC1 function might be hyperactivated in up to 70% of all human tumors; much less information is available for mTORC2, although its link to PI3K/Phosphatase and tensin homolog on chromosome 10 (PTEN) suggests that it is also activated in tumor cells [19,20]. Targeting mTOR signaling has therefore become an attractive strategy in cancer therapy.

Although rapamycin and its analogues (rapalogs) have shown clinical efficacy in a subset of cancer types, they do not fully exploit the potential anti-tumor activity of the mTOR-targeting drugs, to some extent because of their pharmacodynamics. Thus, small molecules that inhibit mTOR kinase activity and dual PI3K/mTOR inhibitors are also being developed. To overcome drug resistance mechanisms developed during single-agent cancer treatment, several combination therapies are currently in clinical trials.

Here, we review current knowledge of mTOR signaling, describe its cross-talk with other pathways, and examine its contribution to human cancer and potential for therapeutic targeting.

## 2. mTOR Complexes and Downstream Effectors

mTORC1 is a multi-protein complex containing several elements that may differ, depending on cell type and localization [1,17]. Raptor is the subunit specific for mTORC1: it is a scaffold protein that facilitates the recruitment of substrates to the mTOR kinase, thus positively regulating its activity [4,6]. Although over time, several authors have shown that Raptor influences exclusively the activity of mTORC1, recent evidence shows that Raptor influenced also the activity of mTORC2: indeed, knocking-down Raptor enhances the activation of mTORC2 [21]. 

PRAS40 is another specific component of mTORC1 and it acts as a negative regulator of mTORC1 by interacting with Raptor and the kinase domain of mTOR [7,22]. PRAS40 is a negative regulator in a dephosphorylated state [23]. Multiple stimuli can trigger the phosphorylation at different sites of PRAS40 and these phosphorylations release the inhibition of mTORC1 by PRAS40: AKT was the first identified kinase, which phosphorylates PRAS40 on Thr246 [24]. 

mTORC1 and mTORC2 share a basic structure with components present and essential to the activity of the complexes, some of which identified in recent years. The effects of these proteins are summarized in Table 1.

DEPTOR is a negative regulator of both mTORC1 and mTORC2 activity in the dephosphorylated state, similarly to PRAS40: indeed, phosphorylation of DEPTOR abolishes its repressive effect toward mTORC1 by reducing its association with mTORC1 [12]. DEPTOR is able to bind mTOR: as opposed to what has been described so far, DEPTOR-mTOR binding inhibits the kinase activity of mTOR [12]. Therefore, the abundance of DEPTOR is critical for the activity of the mTOR network: in response to external signals p70 ribosomal protein S6 Kinase 1 (p70*^S6K1^*) or p90 Ribosomal S6 Kinase 1 (p90*^RSK1^*) phosphorylate DEPTOR, thus resulting in ubiquitination and degradation of the protein [28].

mLST8 binds the kinase domain of mTOR enhancing mTORC1 and mTORC2 activity. mLST8 promotes the activation of the mTOR kinase, but it was demonstrated that it is necessary to maintain the interaction between Rictor and mTOR in mTORC2, and not between Raptor and mTOR in mTORC1 [29]. Kakumoto and colleagues recently demonstrated that mLST8 is more elevated in tissue-specific tumors, such as colon and prostate cancer cell lines, and that mLST8 knock down has no effect on the growth of normal epithelial cells, suggesting that mLST8 plays a marginal role in regulating mTOR function under normal conditions. Moreover, mLST8 knock down induces dissociation of mTORC1/2 complexes in cancer cells and significantly suppresses both mTOR complexes formation, thereby leading to a new role of mLST8 even in mTORC1 [30]. 

In 2010, Kaizuka and colleagues identified for the first time Tti1 as a novel mTOR-binding protein: more specifically, they demonstrated that Tti1 interacts with Tel2, leading to the binding of Tel2 with mTOR. The formation of this complex promotes mTOR stability and activity of mTORC1 and mTORC2, thereby identifying Tti1 as a positive regulator of the stability and activity of both mTOR complexes [25]. Rac1, a member of the Rac subfamily of Rho Guanosil TriphosPhate (GTP)ases, was also identified as a positive regulator of both mTORC1 and mTORC2. Indeed, Rac1 C-terminus directly binds to mTOR and causes its membrane localization, thereby activating both mTOR complexes [10]. mTORC1 and mTORC2 share another component, which, unlike those described above, play their role exclusively in one of the two complexes. GRp58 interacts with mTOR and it is involved in the assembly, but not in the stability, of mTORC1; moreover, GRp58 directly binds the kinase domain of mTOR, thereby enhancing the catalytic activity of mTOR [11].

Two specific positive regulators of mTORC2, both necessary for the stability of the complex, are Rictor and mSIN1, which can reciprocally influence their protein levels. The interaction between Rictor and mSIN1 is very stable in vivo, leading to the hypothesis that they could stabilize each other; indeed, knocking-down mSIN1 decreases the interaction between mTOR and Rictor, thereby regulating mTORC2 stability. On the contrary, Rictor-mSIN1-mTOR is not required for mTORC1 activation, as demonstrated by the activity of p70*^S6K1^* and Elongation Initiation Factor (EIF)-4E Binding Protein 1 (4E-BP1) even in SIN1^−/−^ cells [15].

Two newly identified interactors in mTORC2 are Protor-1 and Protor-2 [23]. Protor-1 and -2 bind specifically to the Rictor subunit of the complex and they are necessary for both mTORC2 assembly and catalytic activity. In 2007, Woo and colleagues identified a novel component of mTORC2, named PRR5: even if PRR5 binds Rictor, it is dispensable for mTOR-Rictor interaction and mTOR activity [26]. Hsp70 is involved in formation and kinase mTORC2 activity under both basal and heat shock conditions [27].

**Figure 2:**
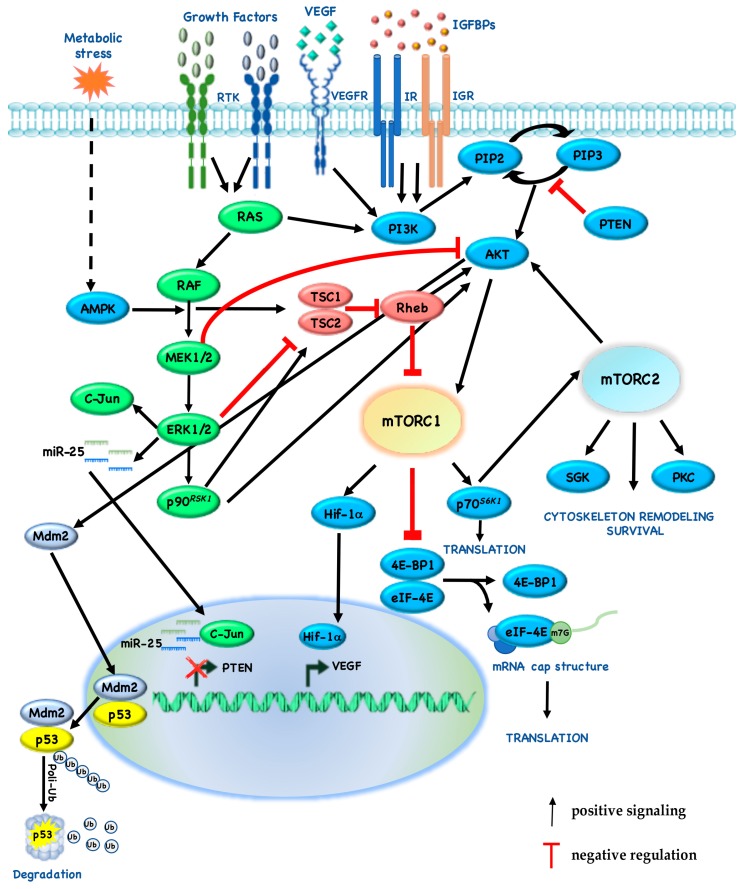
Cross-talk of mTOR with other signaling pathways. The RAS/Mitogen Activated Protein Kinase (MAPK) and phosphoInositide3-Kinase (PI3K)/mTOR pathways respond to extracellular and intracellular stimuli and they extensively cross-talk to both positively and negatively regulate each other. Growth factors bind Receptor Tyrosine Kinases (RTK), which activate both MAPK and PI3K pathway, by regulating a cascade of phosphorylations. Activated MAPK signaling both positively and negatively regulates the activity of members of PI3K/mTOR pathway, by interfering with the assembly of Tuberous Sclerosis Complexes (TSC) 1-TSC2 complex. Activated PI3K phosphorylates PhosphatidylInositol Phosphate (PIP) 2 to generate membrane-bound PIP3, which in turn activates AKT. mTORC1 and mTORC2 activation regulates cell survival, proliferation, motility, angiogenesis, translation and metabolism. Black arrows represent positive signaling, whereas the red ones represent negative regulations.

mTORC1 and mTORC2 are controlled by nutrient levels, growth factors, hormones and hypoxia and, even if they are both involved in the control of glucose metabolism, they have different physiological functions. Indeed, mTORC1 promotes mRNA translation, lipid and nucleotide synthesis and inhibits catabolic processes such as autophagy; in addition to its role in inhibiting apopotosis and promoting cytoscheletal remodeling and cell migration, mTORC2 also has an effect on metabolism (Figure 2) [31].

These two complexes have distinct downstream effectors: 4E-BP1, p70*^S6K1^*, and several key transcription factors are placed downstream of mTORC1, whereas kinases such as AKT, Serum and Glucocorticoid Kinase (SGK) and Protein-Kinase C (PKC) are downstream of mTORC2 [14,16,32,33,34,35]. 

mTORC1-mediated phosphorylation of 4EBP1 and p70*^S6K1^* releases their respective binding partners, eukaryotic translation Initiation Factor (eIF)-4E and eukaryotic Initiation Factor-3 (eIF-3), facilitating translation initiating complex formation and enhancing ribosome biogenesis [36]. Increased cap-dependent translation caused by aberrant mTORC1 activation results in increased proliferation and cell size [37,38]. Both p70*^S6K1^* and 4E-BP1 contain a common mTORC1 Signaling motif (TOS) that is responsible for substrate recognition by Raptor and consequently phosphorylation by mTORC1 [39].

mTORC1 phosphorylates 4E-BP1 at multiple sites to promote the dissociation of eIF-4E from 4E-BP1: free eIF-4E can form eIF-4F complex and this interaction leads to increased translation of mRNAs encoding for proteins required for G1-to-S phase transition. In quiescent cells or under low growth factors levels, low mTOR activity causes 4E-BP1 dephosphorylation, which prevents protein translation [40].

p70*^S6K^* phosphorylates eukaryotic translation Initiation Factor-4B (eIF-4B) and S6 Ribosomal Protein (S6RP), which in turn allows translation and translational elongation. The phosphorylation of eukaryotic Elongation Factor 2 Kinase (eEF-2K) to causes continued translational elongation by eukaryotic Elongation Factor 2 (eEF2) [41,42].

Furthermore, mTORC1 is also involved in the regulation of other proteins including Ornithine DeCarboxylase (ODC), glycogen synthase, Hypoxia-Inducible Factor 1α (HIF-1 α), lipin, Protein Phosphatase 2A (PP2A) and Signal Transducer and Activator of Transcription (STAT) 3 [43,44,45,46,47,48,49]. Through the regulation of these protein sets, mTORC1 promotes the biosynthesis of macromolecules, as well as proteins, lipids, and nucleotides to build the biomass underlying cell, tissue, and organism growth [2].

AKT is a key substrate of mTORC2: its phosphorylation and activation regulates cell growth, survival, and metabolism [34]. Importantly, mTORC2 localization at the cell membrane through the mSIN1 subunit allows mTORC2 to recruit its substrates AKT, SGK, and PKC and this localization is a key aspect of mTORC2 regulation [50]. In response to growth factor stimulation (such as insulin or Insulin Growth Factor (IGF)), sustained phosphorylation of AKT by mTORC2 activity, leads to the phosphorylation and inhibition of TSC2 and this mechanism should in turn upregulate mTORC1 activity [21].

PKCα was the first mTORC2 substrate identified: Jacinto et al. showed that, through PKCα phosphorylation, mTORC2 controls actin cytoskeleton reorganization [14,16]. Recently, it has been demonstrated that mTORC2 phosphorylates other members of PKC family (i.e., PKCδ and PKCε) involved in cytoskeleton remodeling and cell migration [51,52].

mTORC2 also phosphorylates and activates SGK. Substrates of SGK are N-myc Downstream-Regulated Gene 1 protein (NDRG1) and Forkhead box family transcription factors (FoxO), which can promote cell survival in response to oxygen or nutrient deprivation, or in response to PI3K inhibition [53,54].

## 3. Regulators of mTORC1 and mTORC2 Pathway

Activation of the mTORC1 signaling pathway in response to insulin or growth factors occurs primarily through the PI3K/AKT signaling pathway [55]. Growth factors stimulate mTORC1 through the activation of insulin and Ras signaling. The stimulation of these pathways increases the phosphorylation of TSC2 by AKT, Extracellular-signal-Regulated Kinase (ERK) 1/2, and p90*^RSK1^* leading to the inactivation of TSC and thus to the activation of mTORC1 [56,57,58,59]. Indeed, mTORC1 is inhibited by TSC, a heterotrimeric complex composed by TSC1, TSC2 and TBC1 Domain family Member 7 (TBC1D7) [60]. Upon amino acid stimulation, mTORC1 translocates to lysosomes, TSC releases its inhibitory activity on Rheb (a Ras family GTPase which binds mTORC1) thus allowing the activation of mTORC1 [61].

Moreover, AKT activation by growth factors can regulate mTORC1 in a TSC1/2-independent manner through the phosphorylation and dissociation of PRAS40 from mTORC1 [7]. mTORC1 responds to cellular stress such as DNA damage and low levels of both oxygen and Adenosine TriPhosphate (ATP). In DNA damage response mTORC1 activity is reduced. The DNA damage response pathway inhibits mTORC1 through several mechanisms including the induction of p53 target genes, such as 5′-AMP activated protein Kinase β (AMPK β) and PTEN that rapidly increase TSC2 activity [62]. In response to energy depletion, AMPK is activated and inhibits mTORC1 indirectly through phosphorylation and activation of TSC2, and directly by phosphorylation of Raptor [63]. 

Oxygen levels affect mTORC1 activity through multiple pathways, which involve AMPK and DNA Damage and Development 1 (REDD1), as described below [64]. Amino acid levels positively regulate mTORC1 through an amino acid sensing cascade involving Rag GTPases [65]. Amino acid stimulation activates Rag complexes, allowing them to bind Raptor and recruit mTORC1 to the lysosomal surface [66,67].

It was shown that leucine and arginine, two essential amino acids required for mTORC1 activation, signal through a distinct pathway that involved GAP Activity TOwards Rags (GATOR) 1 and 2 complexes [68]. GATOR1 negatively regulates mTORC1, whereas GATOR2 acts as a positive regulator [68]. 

While mTORC1 regulation mechanisms are well characterized, much less is known about upstream regulators of mTORC2. Similar to mTORC1, PI3K is a key modulator of mTORC2: indeed, PI3K promotes the binding of mTORC2 to ribosome thereby activating mTORC2, both in normal and cancer cells [69]. mTORC2 pathway is regulated also by mTORC1, through negative feedback loop between mTORC1 and insulin/PI3K signaling, as demonstrated by some evidence which shows that p70*^S6K1^* promotes the proteasomal degradation of Insulin Receptor Substrate (IRS) 1/2, thus downregulating PI3K/AKT pathway and mTORC2 [70]. Another feedback loop is mediated by Growth factor receptor-bound protein 10 (Grb10), which is positively regulated by mTORC1 and once activated blocks the IGF1 pathway [71,72].

## 4. mTORC1 and mTORC2 and Their Cross-Talk with Other Pathways

The PI3K/AKT/mTOR signaling pathways regulate many biological and physiological processes such as proliferation, survival and angiogenesis, by promoting protein synthesis, glycolysis, lipid biogenesis, metabolism, and by reducing autophagy [2]. Therefore, it is no surprise that elements of the PI3K/AKT/mTOR signaling pathway are among the most frequently mutated genes in tumors [73,74]. The intensity and duration of pathway activation is influenced by both positive and negative feedback loops, which are often involved in multiple signaling cascades, thereby modulating mTOR complexes’ activity. 

### 4.1. Mitogen Activated Protein Kinase (MAPK)

Cross-inhibition between pathways is often revealed when one pathway is chemically blocked, thereby activating the other pathway: for example, Mitogen-activated protein kinase kinase (MEK) inhibitors enhance Epidermal Growth Factor (EGF)-induced AKT activation [75]. On the other hand, AKT negatively regulates ERK activation by phosphorylating inhibitory sites in the Rapidly Accelerated Fibrosarcoma (RAF) N-terminus [76]. Recently, we also identified a novel cross-talk mechanism, by which pharmacologic or genetic inhibition of MEK restores PTEN expression, thus leading to cross-inhibition of downstream signaling through AKT and mTOR: more specifically, ERK-dependent upregulation of c-Jun and miR-25 leads to suppression of PTEN expression (Figure 2) [77]. According to these results, our group demonstrated that, in twenty-nine cancer cell lines with different histological origin (melanoma, n = 7; Breast Cancer (BC), n = 6; Non-Small Cell Lung Cancer (NSCLC), n = 6; ColoRectal Cancer (CRC), n = 8; pancreatic adenocarcinoma, n = 2), PTEN-loss predicts synergistic interaction between MAPK (trametinib, dabrafenib) and PI3K/mTOR (everolimus, MK-2206, gedatolisib) pathway inhibitors, while combined MEK/mTOR inhibition results in a slightly additive/frankly antagonistic growth inhibitory response in PTEN-competent tumor cells (Table 2) [78]. 

Due to the relevance of MAPK-PI3K/mTOR cross-talk in cancer therapeutics, the extensive and dynamic cross-talk between these signaling pathways is now becoming clear. Indeed, during the past years, several mechanisms by which elements of the MAPK pathways can also cross-activate PI3K signaling have been identified: for example, PI3K is activated by RAS and mTORC1 pathway components are phosphorylated by ERK and p90*^RSK1^*. RAS-GTP can directly bind and allosterically activate PI3K [85]. Indeed, ERK activates p90*^RSK1^*, which in turn phosphorylates TSC2 at Ser1798 and AKT at Ser939 and Thr1462 [86]. In addition, ERK phosphorylates Raptor at Ser719/721/722 after mitogen stimulation, thereby promoting mTORC1 activity, 4E-BP phosphorylation and cell growth [87]. ERK1/2 directly phosphorylates TSC1-2 at Ser540 and/or Ser664, thereby disrupting the interaction between TSC1 and TSC2 [59].

### 4.2. Vascular Endothelial Growth Factor (VEGF) and Hypoxia

Angiogenesis is another important factor in cancer progression, with the formation of new blood vessels to provide oxygen and nutrients for cancer cells [88]. We and others have demonstrated that mTOR plays a key role also in angiogenesis, increasing the translation of HIF-1/2, which in turn lead to the expression of hypoxic stress response genes, such as VEGF [43]. More specifically, under normoxic conditions mTORC1 induces HIF-1 α cap-dependent translation through the 4E-BP1–eIF4 axis, thereby increasing the protein levels [89]. Moreover, mTOR inhibitors exert antiangiogenic effects by directly inhibiting endothelial cell functions, such as proliferation and morphogenesis [43].

On the other hand, mTORC1 activity is sensitive to O_2_ deprivation: indeed, in long-lasting hypoxic conditions, hypoxia inhibits mTORC1, by activating the TSC1-TSC2 complex. Many groups have demonstrated several mechanisms by which TSC1-TSC2 complex can block mTORC1 activity. Liu and colleagues demonstrated that hypoxic conditions activate an AMPK-dependent mechanism, which in turn inhibits mTOR and eEF2 [90]. Another study showed that the phosphorylation on Raptor could also influence the activity of the complex: AMPK phosphorylates Raptor on Ser722/792, after energy stress thus leading to the inhibition of mTORC1 [63]. Moreover, De Young and colleagues also showed that REDD1 suppress mTOR activity, by releasing TSC2 from its inhibitory binding with 14-3-3 proteins, in hypoxia [91]. 

The knowledge of a complex relationship between mTOR signaling and hypoxia represents the rationale for combination therapy using inhibitors targeting mTOR and angiogenic factor. 

Recently, Matsuki and collaborators investigated the mechanism at the base of synergistic effect of the lenvatinib plus everolimus combination observed in preclinical xenograft models of Renal Cell Carcinoma (RCC). These studies showed that the combination enhances angiogenesis inhibition and exerts more pronounced direct antitumor effects, as compared to monotherapy, leading to tumor xenograft regression. In particular, the combination enhances inhibition of both Fibroblast Growth Factor (FGF)- and VEGF-induced tumor angiogenesis against endothelial cells, acting on cell proliferation as well as tube formation; moreover, the combination strongly reduces mTOR-S6K-S6 signaling in tumor cells, as compared to monotherapy (Table 2) [80].

### 4.3. Nuclear Factor-κB (NF-κB)

Another master regulator of cancer initiation and progression is NF-κB, a family of five transcription factors, NF-κB1/p105, NF-κB2/p100, RelA/p65, RelB and c-Rel, which can stimulate cell proliferation, angiogenesis, tumor metastasis and metabolism [92]. NF-κB can be activated by loss of tumor suppressors and by oncoproteins, such as AKT which phosphorylates and activates RelA through I Kappa Kinase (IKK) activation [93]. Moreover, IKKα binds mTORC1 complex in a manner dependent on AKT levels, but only in PTEN-loss contexts [94]. IKKβ is also involved in mTORC1 activation: as a consequence of Tumor Necrosis Factor α (TNFα) activation, IKKβ binds and phosphorylates TSC1 at Ser487/511, thereby inhibiting TSC1-TSC2 and activating mTORC1 [95].

Bortezomib exerts antitumor activity against cancer cells, by preventing IKB degradation and therefore inhibiting NF-kB activation [96]. Several studies have demonstrated clinical efficacy of bortezomib and temsirolimus as single agents in Multiple Myeloma (MM) treatments and O’Sullivan and his group demonstrated for the first time that the combination of bortezomib and temsirolimus results in synergistic effects in in vitro MM models [97]. The same synergistic effects were also demonstrated in HepatoCellular Carcinoma (HCC) cell lines, highlighting the relevance of this combination strategy as a novel and promising therapeutic approach (Table 2) [81].

### 4.4. p53

The AKT/mTOR signaling transduction pathway plays a central role in integrating nutrient and growth factor signals, to control cell growth and proliferation, cellular functions that are also crucially controlled by the tumor suppressor p53. Therefore, it is not surprising that a reciprocal regulation between PI3K/AKT and p53 (and its specific E3 ubiquitin ligase, Mouse double minute 2 homolog (Mdm2)) pathways exists. Indeed, Demidenko and collaborators have demonstrated that p53 can suppress cellular senescence and in quiescent cells p53 inhibits the pro-senescence effects of the mTOR pathway [98,99].

Moreover, p53 directly binds a region of the PTEN promoter, upregulating PTEN expression and hampering PI3K/AKT/mTOR activity [100]. On the other hand, PI3K and its direct downstream target AKT phosphorylate Mdm2 at Ser166/186, thus leading to the translocation of Mdm2 in the nucleus and inhibition of p53 activity [101]. Goudarzi and collaborators have investigated the outcomes of simultaneous inhibition of mTOR and activation of p53 by nucleolar stress, which is triggered by chemotherapeutic drugs: they demonstrated that rapamycin increased the levels of endogenous Mdm2 despite inhibition of its phosphorylation at Ser166, underlying the complexity of the interplay between p53 and mTOR in cancer [102].

Activation of PI3K/mTOR pathway and overexpression of Mdm2 are frequent molecular features in Acute Myeloid Leukemia (AML): according to the interactions between PI3K/mTOR and Mdm2/p53 pathways, Kojima and colleagues demonstrated that treatment with PI-103 (a dual PI3K/mTOR inhibitor) enhances p53 downstream signaling in p53 wild-type AML contexts [82]. Moreover, the same group showed that a simultaneous inhibition of the PI3K/mTOR pathway and Mdm2-p53 complex assembly (by nutlin-3) is potentially effective in AML (Table 2) [82]. Even in mesothelioma, p53 pathway is often defective, thus leading to conventional treatments resistance, and Shimazu and his group investigated a possible mechanism of the combinatory effects between up-regulation of p53 levels and inhibition of the mTOR pathways, by nutlin-3 and metformin respectively [103]. Nevertheless, in this work the authors demonstrated that combination of metformin and nutlin-3a results in both additive and synergistic effects, probably due to heterogeneity of mesothelioma cells. These results point to the need to elucidate the mechanism of action by which this drug combination may be synergistic.

### 4.5. Epigenetics

During the last years, new evidence identified the presence of abnormal DNA methylation patterns in cancer cells: therefore, specific chemical inhibitors are now being developed to target tumors with mutations in these genes, such as Histone DeACetylase (HDAC) and Histone MethylTransferase (HMT) inhibitors [104,105].

Several studies pointed to potential drug combinations between mTOR inhibitors and epigenetic drugs, in order to overcome both innate and acquired drug resistance. Simmons and his colleagues demonstrated synergistic interactions between rapamycin and entinostat (small molecule inhibitor of Class 1 HDAC) in B cells neoplasia, in both in vitro and in vivo models (Table 2) [83]. Subsequently, the same group demonstrated that this combination is synergistic in other cancer cell lines with different histological origin (i.e., BC and plasmacytoma) and hypothesized that Myc could be a central player in this combination strategy [106]. Combination approaches include different epigenetic drugs, such as azacitidine, a chemical analog of cytidine which inhibits DNA methyltransferase, causing hypomethylation of DNA; a phase Ib/II study to assess the effects of everolimus in combination with azacitidine in AML patients has been reported [107]. Due to the positive outcome of these studies, the combination strategies with mTOR inhibitors and epigenetic drugs could be an innovative clinical approach in cancer therapy.

### 4.6. STAT and Immune System

**Figure 3:**
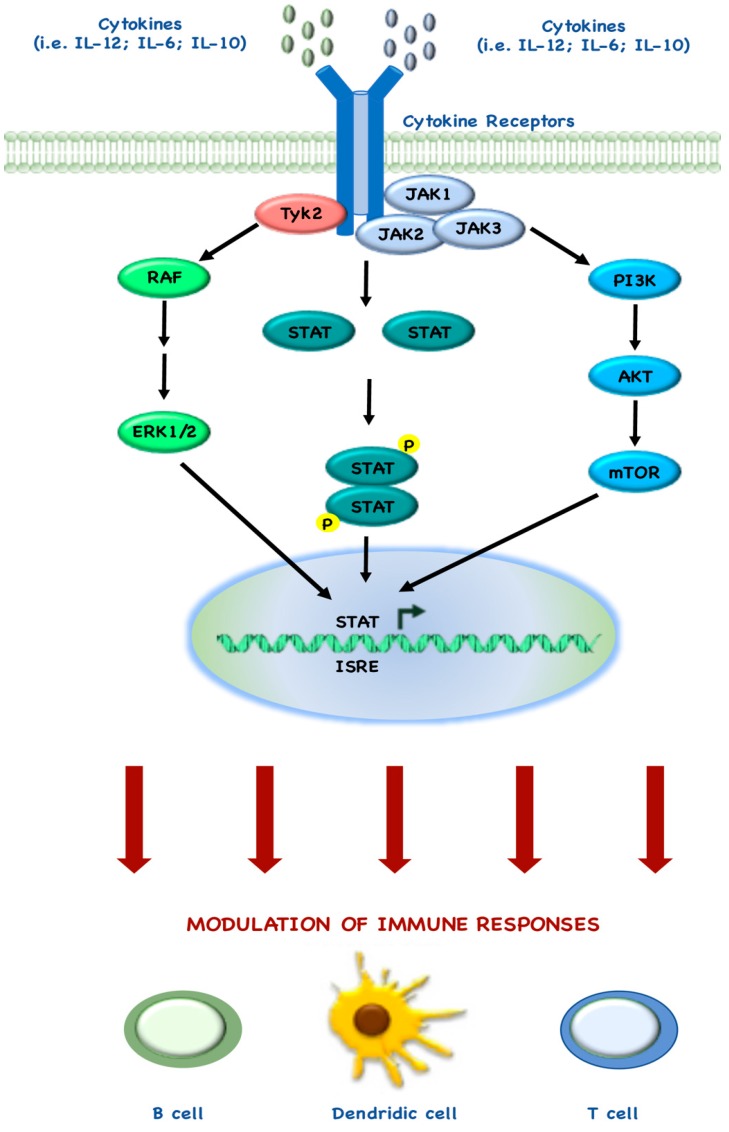
mTOR and STAT signaling interaction in immune response. Cytokines bind trans-membrane receptors, which activate intracellular signaling, such as MAPK, STAT and PI3K/mTOR. These pathways cross-regulate each other to modulate the activity of immune cells.

Several studies also describe the mechanisms behind the tight connection between mTOR and immune regulatory targets, as demonstrated by the Food and Drug Administration (FDA) approval of rapamycin as an immunosuppressive drug, due its ability to block T cell activation [108]. Indeed, during the past few years, many groups have demonstrated how mTOR regulates immune cell homeostasis, both in adaptive and innate immune cells and in recent years there has been accumulating evidence that STAT pathways also play a central role in immune responses, by interacting with the elements of mTOR pathway (Figure 3) [109].

Rao and colleagues demonstrated that InterLeukin (IL)-12 drives the maturation of naïve CD8^+^ T cells, by synergistic co-regulation of PI3K and STAT4: indeed, rapamycin treatment during IL-12 stimulation, blocks the T cell differentiation leading to memory T cell differentiation [110]. Similar to these findings, Li and colleagues also showed that the balance between mTOR and STAT5 may be required for optimal immune response: indeed, they demonstrated that IL-15 is involved in the mechanism behind mTOR-induced CD8^+^ cell differentiation, through STAT5 phosphorylation [109,111]. Other groups investigated how IL-6 and STAT3 are involved in CD4^+^ T cell differentiation in Th17: for example, Yoshimura and colleagues demonstrated that mTORC1 activates STAT3, which in turn promotes Th17 differentiation [112]. Monocytes and macrophages represent one of the most numerous populations of infiltrating tumor cells and they play an active and central role in immune responses. Even in the development and function of Dendritic Cells (DCs), mTOR and STAT3 cross-regulate each other, underlying the complex cross-talk between these pathways. In particular, two different groups independently demonstrated that rapamycin inhibits IL-10 expression and STAT3 phosphorylation in LipoPolySaccharide (LPS)-stimulated DCs [113,114]. Conversely, more recently another group demonstrated that rapamycin does not affect IL-10/IL-10 receptor-mediated activation of STAT3, thereby leading to the hypothesis that mTORC1 activity seems to indirectly control STAT3 activation by regulating IL-10 production in LPS-stimulated DCs [115]. 

The evidence of this complex network in mTOR-STAT cross-talk in the immune system has led to the development of combination therapies with mTOR and JAnus Kinase (JAK)/STAT inhibitors. For example, Bogani and her group investigated the effects of mTOR inhibitors in combination with JAK2 inhibitors, in in vitro MyeloProliferative Neoplasms (MPN) cells [116]. More recently, Miyata and colleagues investigated the effect of a combination of the STAT3 inhibitor (STX-0119) and mTOR inhibitor (rapamycin) in in vitro temozolomide-resistant glioblastoma models (Table 2) [84]. Further studies are needed in order to precisely define elements involved in cross-regulation between the two pathways.

## 5. mTOR and Cancer

Given the central role of mTOR signaling in regulating fundamental biological events, there is a predictable association between mTOR pathway activation and cancer [117]. Mutations in the mTOR gene, that render it constitutively active, have been identified in a few human cancers, even under nutrient starvation conditions, although there are not clearly linked to tumor development [118]. More recently, a detailed analysis of cancer-associated mTOR mutations, derived from publicly available tumor genome sequencing datasets, was conducted by Grabiner et al. [119]. Thirty-three mutations were included in such comprehensive catalogue, clustering in six distinct regions in the C-terminal half of the mTOR protein [119]. Mutations occurred in multiple cancer types, with one of the reported clusters being particularly prominent in kidney cancer and conferring pathway hyperactivation and, potentially, sensitivity to targeted therapies directed against mTOR. Additionally, recurrent genetic aberrations in specific components of the two distinct mTOR complexes have been reported in association with cancer. Amplification of Rictor was observed in NSCLC and BC patients [120,121,122]. More recently, we have reported on Rictor amplification in resected squamous cell carcinoma of the lung, where Rictor amplification is found almost exclusively in patients with poor prognosis and short disease-free survival (27.3% vs. 3.7% in patients with poor and good prognosis, respectively, *p* = 0.017) [123].

However, activation of the mTOR pathway can also result from mutations in either upstream class of genes: tumor suppressor and oncogenes depending on whether they activate or suppress pathway signaling. Different elements of the PI3K signaling pathway, which is upstream of both mTORC1 and mTORC2 are mutated in human cancers [65]. Frequently occurring mutations that activate the PI3K-AKT pathway in cancer include AKT mutation and amplification, PIK3CA-activating mutations and gene amplification, and growth factor receptors amplification (i.e., Epidermal Growth Factor Receptor (EGFR), Insulin Growth Factor Receptor (IGFR), IRS) [50,124,125]. Moreover, loss of tumor suppressors, such as PTEN, are involved in hyperactivation of the PI3K/mTOR pathway. PTEN expression is downregulated in many human cancers through several mechanisms which include mutation, Loss Of Heterozygosity (LOH), protein instability, methylation and cellular sublocalization [126]. Interestingly, PTEN mutations are associated with sensitization to mTOR inhibitors in myeloma, breast and endometrial cancer cells [127,128,129,130]. Mutations in TSC1/TSC2, p53, and STK11, all negative regulators of mTOR, can also result in mTOR activation [131,132].

mTOR activation may play a role in several aspects of tumor initiation and progression. Increasing evidence shows that deregulation of protein synthesis downstream of mTORC1 at the level of 4E-BP1/eIF-4E may be involved in cancer initiation [1]. mTOR regulates anabolic metabolism thought different mechanisms, including the de novo purine and pyrimidine synthesis and lipogenic gene expression [89,133,134]. For example, PI3K signaling promotes the activation of the pro-lipogenic factor Sterol Regulatory Element-Binding Protein 1 (SRE-BP1), a key transcriptional factor that controls lipogenesis and lipid uptake, through AKT activation in a manner dependent on mTORC1 and lipid synthesis is a hallmark of proliferating cancer cells [89,135]. mTOR is also a central regulator of glucose metabolism, facilitating the incorporation of nutrients into new biomass. More specifically, mTORC1 increases the translation of two critical transcription factors, Myc and HIF-1 α, which drive the expression of several glycolytic enzymes, such as Phospho-Fructo Kinase (PFK); on the other hand, the downstream effector of mTORC2, AKT, once activated increases the rate of glucose metabolism [89,136,137].

mTOR is involved in inhibition of autophagy, which is activated in response to nutrient stress and energy deficiency, both under physiological and pathological conditions; once activated, autophagic processes activate lysosomal degradation of cytosolic components. Recently, three groups independently demonstrated that mTORC1 phosphorylates UNC-5 Like autophagy activating Kinase (ULK) 1/2, leading to its inactivation. Indeed, when activated, ULK1/2 phosphorylates Autophagy Related Gene (ATG) 13 and FIP200, thereby activating autophagic processes [138,139,140]. Autophagy is regulated by mTORC1 also at the transcriptional level, modulating localization of Transcription Factor EB (TFEB), which is a master transcriptional factor of lysosomal and autophagy genes [141]. Autophagy, in turn, may have a dual role in tumor development: indeed, in a recent study by Rosenfeldt et al. in a transgenic model of Pancreatic Ductal Adenoma Carcinoma (PDAC), inhibition of autophagy blocked the progression of low-grade pancreatic intraepithelial neoplastic lesions to high-grade precancerous lesions and frank cancer in mice with wild-type p53, whereas its inhibition in the presence of p53 mutations promoted cancer formation [142,143].

Quite interestingly, while contributing to cancer initiation and progression, prolonged mTOR stimulation in normal cells can paradoxically lead to stem cell depletion, reduced health and lifespan [144]. Constitutive AKT activation or PTEN-loss deplete normal Hematopoietic Stem Cells (HSCs) and induces leukemia in mice [145,146,147]. These effects are dependent on mTOR activation, as they can be inhibited by rapamycin, which not only depletes leukemia-initiating cells but also restores normal HSCs function [148]. TSC1 deletion also induces rapid HSCs cycling and depletion, in an mTOR-dependent manner [149]. mTORC1 regulates stem cell self-renewal in epithelial cells, as well; indeed, prolonged mTOR signaling induces adult epidermal stem cell exhaustion and hair loss in mice, both of which are delayed by rapamycin treatment [150]. Additionally, caloric restriction inhibits mTORC1 activity, thereby increasing intestinal stem cell numbers [151]. Finally, mTOR is also involved in the tumorigenesis process by altering the tumor microenvironment. Indeed, in response to oxygen and/or nutrient deprivation, mTOR stimulates tumor cells to secrete factors, such as VEGF, that recruit new vessel formation to support the tumor growth [43,152]. In addition, mTOR controls actin cytoskeleton remodeling and cell motility through regulation of the expression of small GTPase such as RhoA, Rac1 and Cdc42 [152,153,154].

## 6. Clinical Development of mTOR Pathway Inhibitors

Due to the central role played by the mTOR pathway in many aspects of carcinogenesis and tumor progression, considerable effort was put in biological investigations focused on targeting and switching-off hyperactivated mTOR signaling in cancer cells, thereby leading to the development of several inhibitors for cancer treatment.

Rapamycin, an antifungal agent with immunosuppressive properties, was the first mTOR inhibitor developed for anti-cancer activity in the 90s. Rapamycin does not directly inhibit the catalytic activity of mTOR, but it binds to FK506 Binding Protein 12 (FKBP12) in a complex that allosterically inhibits the FKBP12-Rapamycin Binding (FRB) domain of mTORC1, thus leading to the dissociation of Raptor from mTORC1. The inhibition of mTORC1 downstream signaling is detected by suppression of p70*^S6K1^* and 4E-BP1 phosphorylation and the inactivation of these proteins results in a reduction of protein translation and synthesis and cell cycle arrest in the G1 phase [155]. As mTORC1 activation increases HIF-1 α levels, by stimulating the translation of its mRNA, rapamycin downregulates several HIF transcriptional targets, such as VEGF, Platelet-Derived Growth Factor (PDGF), basic Fibroblast Growth Factor (bFGF) and other growth factors involved in angiogenesis and tumor progression [156]. Although rapamycin does not interact with mTORC2, it can affect mTORC2 indirectly: by binding to mTOR as a complex with FKBP12, it prevents mTOR from associating with Rictor, therefore causing a downregulation in mTORC2 levels [157,158]. Moreover, Lamming and colleagues demonstrated that rapamycin treatment induces insulin resistance in mice, due the role of mTORC2 in insulin-mediated suppression of hepatic gluconeogenesis [159]. 

Even though the antineoplastic properties of rapamycin have been extensively documented in several tumors, its development as an anti-cancer agent was hampered by poor solubility and a relatively unpredictable pharmacokinetic profile. For this reason, several rapamycin analogs, collectively referred to as *rapalogs*, were developed: they all have similar structures and mechanisms of action, but different pharmacokinetic profiles. To date, three rapalogs (one intravenous agent—temsirolimus—and two oral agents—everolimus and ridaforolimus) have been tested in phase III clinical trials in different cancers, as either monotherapy or in combination with other agents (Table 3). 

### 6.1. Monotherapy with Rapalogs

Currently, two rapalogs, temsirolimus and everolimus, are approved in US and EU for the treatment of different types of cancer. A third agent—ridaforolimus, was tested as maintenance therapy in sarcoma patients achieving disease control with chemotherapy in the phase III SUCCEED trial (Table 3); although the trial was formally positive, in that it met its primary endpoint of reducing the risk of progression or death by at least 25% (Hazard Ratio (HR) for Progression-Free Survival (PFS), 0.72; 95% Confidence Interval (CI), 0.61–0.85; *p* = 0.001, the extent of PFS benefit (3 weeks in median: 17.7 vs. 14.6 weeks) was felt to be too small in the absence of a concomitant Overall Survival (OS) benefit to warrant regulatory approval and further development of the drug) [165]. Conversely, temsirolimus monotherapy is approved for the first-line treatment of metastatic RCC (mRCC) with *poor risk* features based on the results of the ARCC trial, and for the treatment of relapsed/refractory Mantle Cell Lymphoma (MCL) (see Table 3); everolimus monotherapy, on the other hand, is approved for the treatment of advanced, pretreated mRCC and progressive NeuroEndocrine Tumors (NET) of Gastro-Entero-Pancreatic (GEP) and lung origin, based on the RADIANT-3 and -4 trials (Table 3) [160,163,164,166]. 

Rapalogs have been extensively evaluated for the treatment of many other tumor types, but such investigations have met with very limited clinical success, despite the fact that the PI3K/AKT/mTOR pathway is frequently dysregulated in human cancers and it plays a fundamental biological role as a master regulator of cell growth and proliferation, cellular metabolism, and cell survival [174]. This might be due, at least in part, to the fact that clinical trials with mTOR inhibitors have been conducted in unselected patient populations, without enrichment for potential biomarkers of sensitivity or predictors of clinical activity (see Section 6.4). 

Rapalogs monotherapy is in general well tolerated and adverse events are manageable and related to their mechanism of action; their description is beyond the scope of this review, but are well described in the literature [175,176,177,178].

### 6.2. mTOR Kinase Inhibitors 

The limited clinical success of rapalogs may be related to feedback loops involved in cell survival responses. For instance, under normal conditions, mTORC1 activates p70*^S6K1^* which phosphorylates IRS, thereby leading to its degradation and downregulation of PI3K signaling: rapamycin treatment blocks mTORC1 activity, IRS is activated and PI3K signaling is upregulated [179]. Rapalogs can also cause activation of AKT through disruption of a negative feedback loop on the mTORC2 complex, which is involved in cancer cell growth and survival: this limitation led to development of a second generation of mTOR inhibitors, which are ATP-competitive mTOR kinase inhibitors [179]. These compounds suppress the activation of both mTORC1 and mTORC2, thus completely blocking PI3K/AKT signaling and prevent the feedback activation of AKT after treatment with rapalogs. Due to the sequence similarity between mTOR and PI3K, particularly at the kinase active sites, ATP-competitive inhibitors often inhibit both PI3K and mTOR activity and are therefore referred to as dual inhibitors [180]. Several dual PI3K/mTOR inhibitors (e.g., GDC-0980, PF-04691502, BEZ235, XL765, GSK2126458) are currently being developed for clinical use, on the assumption that the vertical blockade of two different crucial nodes along the PI3K signaling pathway might result in more complete pathway inhibition, disruption of pathway-reactivating feedback loops, and eventually enhanced anti-tumor activity [180,181]. However, limited clinical experience obtained so far suggests that such agents have only modest single agent anti-tumor activity. This may partly be due to the narrow therapeutic window associated with these drugs that limits their dose escalation, or to the unselected populations of patients enrolled into these early phase studies (see Section 6.4 and Section 6.5) [181,182]. Alternatively, clinically meaningful mTOR pathway blockade could be achieved by non-ATP-competitive allosteric modulators of protein functions and possibly by Hsp90 inhibitors (reviewed in [183]).

### 6.3. Third-Generation mTOR Inhibitors

Based on evidence that mutations in either the FRB or the kinase domain can induce resistance to rapalogs and mTOR kinase inhibitors, third-generation mTOR inhibitors exploiting the unique juxtaposition of two drug (first- and second-generation mTOR kinase inhibitors)—binding pockets to create a bivalent interaction that allows inhibition of the mutants have been developed [184,185,186]. Rapalink-1 is more potent than first- and second- generation mTOR inhibitors in reducing the levels of both p-4EBP1 and cell proliferation; as a consequence, RapaLink-1 led to regression of tumor xenografts models and could durably block mTORC1; moreover, it showed better efficacy than rapamycin or mTOR kinase inhibitors, potently blocking cancer-derived, activating mutants of mTOR. No clinical data on third-generation mTOR inhibitors are available to date.

### 6.4. Potential Biomarkers of Sensitivity to mTOR Pathway Inhibitors

Several preclinical studies have suggested that alterations in certain tumor suppressor genes (namely PTEN and TSC1/2), as well as mTORC1 phosphorylation sites on 4E-BP1 and p70*^S6K1^* may be correlated with sensitivity or resistance to rapalogs [187]. Moreover, other markers of upstream and downstream signaling have been evaluated to help predict clinical responses [188,189,190]. From a clinical point of view, a recent analysis of 39 patients with (n = 22) or without (n = 17) exceptional clinical benefit from everolimus treatment across various tumor types (13 gastric cancers, 15 RCCs, 2 thyroid cancers, 2 head and neck cancer, and 7 sarcomas) reported mutations in genes along the mTOR pathway (*mTOR*, *TSC1*, *TSC2*, NeuroFibromin *(NF) 1*, PhosphIinositide-3-Kinase Catalytic (*PIK3C) A* and *G)* in 10/22 responder patients (45%), with mutations in *mTOR*, *TSC1/2*, and *NF1* exclusively found in responders; conversely, recurrently mutated genes of Fibroblast Growth Factor Receptor (*FGFR) 4* and BRCA1-Associated Protein *(BAP) 1* were noted only in patients without clinical benefit [191]. 

In a prospective clinical trial conducted in patients with several different advanced/refractory malignancies subjected to extensive molecular profiling and everolimus treatment, only loss-of-function aberrations in *PTEN* significantly correlated with clinical benefit; it should be noted, however, that these *PTEN* aberrations often coincided with other mTOR pathway-related mutations [192]. In the retrospective analysis of a randomized trial comparing everolimus with a VEGF Receptor (VEGFR)-Tyrosine Kinase Inhibitor (TKI) (sunitinib) as first-line treatment for mRCC (RECORD-3 trial), Protein PolyBroMo-1 (*PBRM1*) and *BAP1* mutations were associated with significantly longer (median 12.8 vs. 5.5 months) and shorter (median 4.9 vs. 10.5 months) PFS, respectively, in patients undergoing everolimus treatment [193]. 

Recently, an exceptional response to everolimus was reported in a metastatic BC patient, whose tumor harbored a F354L point mutation in *STK11* [194]. In an exploratory analysis of the BOLERO-2 and BOLERO-3 studies, everolimus was associated with a decreased hazard of progression in patients with *PIK3CA* mutations (HR 0.67; 95% CI, 0.45 to 1.00), PTEN-loss (HR, 0.54; 95% CI, 0.31 to 0.96), or hyperactive PI3K pathway (HR, 0.67; 95% CI, 0.48 to 0.93), while patients with wild-type *PIK3CA*, normal PTEN, or normal PI3K pathway activity did not derive PFS benefit from everolimus [195]. Perhaps the most solid association between potentially predictive molecular alterations and exquisite sensitivity to mTOR inhibitor treatment is found with TSC-loss. Indeed, mutations in either *TSC1* or *TSC2* have been found in patients with bladder, anaplastic thyroid, hepatocellular, and kidney cancer and exceptional clinical responses to rapalogs [185,196,197,198]. Such association is strengthened by the observation that rapalogs have proven effective in ameliorating several signs and symptoms of the TSC complex (an autosomal dominant genetic disorder, belonging to the group of neuro-cutaneous syndromes, characterized by loss-of-function mutations in *TSC1* or *TSC2* genes), including the formation of tumors (such as subependymal giant cell astrocytomas, renal angiomyolipomas, cardiac rhabdomyomas, etc.) [199,200]. Indeed, everolimus is approved by the FDA for the treatment of subependymal giant cell astrocytomas and renal angiomyolipomas associated with the TSC complex. Evidence derived from individual cases and/or limited series has triggered the design of a currently ongoing *basket* trial in which patients with histologically confirmed, advanced/refractory malignancies, harboring confirmed inactivating mutations in *TSC1* or *TSC2*, or activating mutations in *mTOR*, are prospectively treated with everolimus (NCT02201212). 

### 6.5. Prospects for Combination Therapy

Since mTOR signaling confers resistance to several targeted cancer therapies, in recent years focus has shifted towards the development of mTOR-inhibition based combination therapies [201]. Toxicity issues and the necessity to identify reliable biomarkers to select patients at the highest chance of benefit are of paramount importance for the successful development of combination strategies in a clinical setting. Indeed, combinations of targeted agents, including those involving mTOR inhibitors, are usually more toxic than monotherapy alternatives, requiring the administration of grossly reduced single-agent doses, thus potentially compromising anti-tumor activity; on the other hand, the functional effects of a given combination may either be synergistic or antagonistic, depending on the specific molecular context of the individual tumor. As an example, combined MEK/mTOR inhibition has shown substantial clinical toxicity in a recently completed phase I study, where a recommended phase II dose and schedule of trametinib in combination with everolimus could not be identified; however, durable disease control was observed in approximately 30% of patients, suggesting that some patients may derive clinically significant benefit, even if treated with largely suboptimal single-agent doses [202]. Our own preclinical data suggest that PTEN status may potentially be developed as one such biomarker of clinical situations in which combined inhibition of the MEK/ERK and PI3K/AKT/mTOR pathways could be highly synergistic and require reduced single-agent doses of each agent, thereby reducing toxicity [78]. On the other hand, a combination of MEK and AKT inhibitors (selumetinib/MK-2206) has recently been added to a long list of targeted agents that have failed clinical testing in advanced PDAC; given the rare occurrence of inactivating PTEN point mutations or LOH in human PDAC, the failure of selumetinib/MK-2206 to achieve clinical benefit in unselected PDAC patients would have been largely anticipated, based on preclinical data showing lack of PTEN dysfunction and of growth inhibitory synergism with that particular combination of agents [79,203]. 

As highlighted above, a strong rationale exists for combined targeting of the VEGF/VEGFR axis and mTOR pathway; this is particularly true for mRCC, which often shows bi-allelic loss of the von Hippel-Lindau (VHL) gene and increased production of HIF-1 α, correlating with a significant increase in VEGF production; such evidence underlies the rationale of further investigating the molecular mechanisms responsible of the combination therapy effects [204]. Moreover, VEGF/VEGFR and mTOR targeting, as single treatments and in different lines of therapy, represent the mainstays of clinical mRCC treatment. Despite the failure of earlier attempts at combining VEGF/VEGFR- and mTOR-targeting agents, due to a mix of toxicity issues and lack of efficacy, a recent phase II randomized trial has established the combination of lenvatinib (an oral multitarget tyrosine kinase inhibitor of VEGFR-1, -2 and -3, with additional inhibitory activity against FGFR 1-4, PDGFRα, REarranged during Transfection (RET), and KIT) and everolimus, as one of the standards of care for the II-line treatment of advanced RCC [168,173,205,206]. In this trial, Motzer et al. randomized mRCC patients progressing after a previous VEGFR-targeted treatment to either lenvatinib monotherapy, everolimus monotherapy, or their combination at reduced doses; overall, lenvatinib plus everolimus and lenvatinib alone resulted in a significant PFS benefit, as compared to everolimus monotherapy; PFS in the combination group was almost double in comparison to the lenvatinib alone group, although this difference did not reach statistical significance. Together with the results of trials that have established the combination of everolimus and exemestane as a new standard for the treatment of hormone-dependent BC, these data provide a strong proof-of-concept of the feasibility and efficacy of mTOR-inhibition based combination strategies.

## 7. Conclusions 

mTOR plays a crucial role in normal physiology and in various diseases, including cancer; thus, understanding how mTOR signaling pathways work and developing agents that would interfere with its signaling for therapeutic purposes have been a main focus of research in the past 20 years. Despite significant clinical successes, that have led to the use of mTOR inhibitors, both as monotherapy and in synergistic combinations with other agents, in the treatment of several human cancers and genetic conditions, such as the TSC complex, many aspects of the mTOR pathway still remain to be explored. The identification of candidate inhibitors with novel mechanisms of action, the recognition of prognostic/predictive biomarkers, and the modeling and testing of rational, mTOR inhibition-based, combinatorial strategies endowed with highly synergistic anti-tumor activity will pave the way for a new generation of effective and personalized cancer treatments.

## Figures and Tables

**Table 1:** Function of mTOR complexes elements.

mTOR Complex Component	mTORC1 (1) and mTORC2 (2)	Mode of Action	References
Raptor	1	Positive regulator	[4,5,6]
PRAS40	1	Negative regulator	[7,8,9]
DEPTOR	1–2	Negative regulator	[12]
mLST8	1–2	Positive regulator	[5]
Tti1 and Tel2	1–2	Positive regulator	[25]
Rac1	1–2	Positive regulator	[10]
GRp58	1–2	Positive regulator	[11]
Rictor	2	Positive regulator	[13,16]
mSIN1	2	Positive regulator	[15]
Protor 1/2	2	Positive regulator	[23]
PRR5	2	NA	[26]
Hsp70	2	Positive regulator	[27]

NA, Not Available.

**Table 2:** Combination and proposed targets.

I Drug	Target	II Drug	Target	Effect of Interaction	References	
Everolimus; MK-2206; gedatolisib	mTOR; AKT; PI3K/mTOR	trametinib; dabrafenib	MEK; BRAF	Synergism in PTEN-loss context		[78,79]
Everolimus	mTOR	lenvatinib	VEGFR	Synergism in FGF-activated endothelial cells		[80]
Rapamycin	mTOR	bortezomib	IKB	Downregulation of AKT phosphorylation		[81]
PI-103	PI3K/mTOR	nutlin-3	Mdm2	Apoptosis in p53-wt context		[82]
Rapamycin	mTOR	entinostat	DNA methyltransferase	Enhanced cell cycle arrest and apoptosis		[83]
Rapamycin	mTOR	STX-0119	STAT3	Regulation of YKL-40 expression		[84]

MEK, Mitogen-activated protein kinase kinase; RAF, Rapidly Accelerated Fibrosarcoma; PTEN, Phosphatase and tensin homolog on chromosome 10; VEGFR, VEGF Receptor; FGF, Fibroblast Growth Factor; Mdm2, Mouse double minute 2 homolog; STAT, Signal Transducer and Activator of Transcription; YKL, chitinase 3-like protein; wt, wild-type.

**Table 3:** Completed phase III trials with mTOR inhibitors.

Trial	Study Details	Disease	mTOR Inhibitors	Primary Endpoint	References
ARCC	Randomized, open label	RCC	Tem vs. IFN	OS; (HR 0.73; 95% CI 0.58–0.92; *p* = 0.008)	[160]
RECORD-1	Randomized double blind, placebo controlled	RCC	Eve vs. BSC	PFS; HR 0.30, 95% CI 0.22–0.40, *p* < 0.0001	[161]
INTORSECT	Randomized, open label	RCC	Tem vs. Soraf	PFS; HR 0.87, 95% CI 0.71–1.07, *p* = 0.19	[162]
RADIANT-3	Randomized double blind, placebo controlled	PNET	Eve vs. BSC	PFS; HR 0.35, 95% CI 0.27–0.45, *p* < 0.001	[163]
RADIANT-4	Randomized double blind, placebo controlled	Lung/GEP NET	Eve vs. BSC	PFS; HR 0.48, 95% CI 0.35–0.67, *p* < 0.00001	[164]
SUCCEED	Randomized double blind, placebo controlled	Sarcoma	Rida vs. BSC	PFS; HR 0.72, 95% CI 0.61–0.85, *p* = 0.001	[165]
Mantle cell lymphoma	Randomized, open label	MCL	Tem vs. IC	PFS; HR 0.44, 95% CI 0.25–0.78, *p* = 0.0009	[166]
GRANITE-1	Randomized double blind, placebo controlled	Gastric	Eve vs. BSC	OS; HR 0.90, 95% CI 0.75–1.08, *p* = 0.124	[167]
INTORACT	Randomized, open label	RCC	Tem + Beva vs. IFN + Beva	PFS; HR 1.1, 95% CI 0.9–1.3, *p* = 0.8	[168]
RADIANT-2	Randomized double blind, placebo controlled	NET	Eve + Oct vs. Oct	PFS; HR 0.77, 95% CI 0.59–1.00, *p* = 0.026	[169]
BOLERO-2	Randomized double blind, placebo controlled	BC	Eve + Exe vs. Exe	PFS; HR 0.43, 95% CI 0.35–0.54, *p* < 0.001	[170]
BOLERO-3	Randomized double blind, placebo controlled	BC	Eve + Vnr + Trast vs. Vnr + Trast	PFS; HR 0.78, 95% CI 0.65–0.95, *p* = 0.0067 *	[171]
HORIZON	Randomized double blind, placebo controlled	BC	Tem + Letro vs. Letro	PFS; HR 0.90, 95% CI 0.76–1.07, *p* = 0.25	[172]
NCT01136733 ^§^	Randomized phase II study	mRCC	Lenv + Eve vs. Lenv. vs. Eve.	PFS; HR 0.40, 95% CI 0.24–0.68, *p* = 0.0005	[173]

Tem, Temsirolimus; IFN, Interferon; Beva, bevacizumab; Eve, everolimus; Soraf, sorafenib; Oct, octreotide; Rida, ridaforolimus; Exe, exemestane; Letro, letrozole; Vnr, vinorelbine; Trast, trastuzumab; IC, Investigator Choice; BSC, Best Supportive Care; Lenv, lenvatinib; mRCC, metastatic Renal Cell Carcinoma; GEP, Gastro-Entero-Pancreatic; PNET, Pancreatic NeuroEndocrine Tumor; BC, Brest Cancer; HR, Hazard Ratio; CI, Confidence Interval; OS, Overall Survival; PFS, Progression Free Survival; * Everolimus has not been approved for this indication; ^§^ number registration available at ClinicalTrials.gov.

## Abbreviations

The following abbreviations are used in this manuscript:

AML	Akute Myeloma Leukemia
AMPK-β	5′-AMP activated protein Kinase β
ATG	Autophagy Related Gene
ATP	Adenosine TriPhosphate
BAP	BRCA1-Associated Protein
BC	Breast Cancer
bFGF	basic Fibroblast Growth Factor
CI	Confidence Interval
CRC	ColoRectal Cancer
DEPTOR	DEP domain-containing mTOR interacting protein
eEF-2K	eukaryotic Elongation Factor 2 Kinase
eEF2	Elongation Factor 2
EGF	Epidermal Growth Factor
EGFR	Epidermal Growth Factor Receptor
eIF-3	eukaryotic Initiation Factor-3
eIF-4B	eukaryotic translation Initiation Factor-4B
eIF-4E	eukaryotic translation Initiation Factor-4E
eIF-4F	eukaryotic translation Initiation Factor-4F
ERK	Extracellular-signal-Regulated Kinase
FDA	Food and Drug Administration
FGF	Fibroblast Growth Factor
FGFR	Fibroblast Growth Factor Recptor
FKBP12	FK506 Binding Protein 12
FRB	FKBP12-Rapamycin Binding
FoxO	Forkhead box family transcription factors
GATOR	GAP Activity TOwards Rags
GEP	Gastro-Entero-Pancreatic
Grb10	Growth factor receptor-bound protein 10
GTP	Guanosil TriphosPhate
HCC	HepatoCellular Carcinoma
HDAC	Histone DeACetylase
HIF-1	hypoxia-inducible factor 1
HMT	Histone MethylTransferase
HR	Hazard Ratio
HSCs	Hematopoietic Stem Cells
Hsp-70	Heat shock protein 70-α
IGF	Insulin Growth Factor
IGFR	Insulin Growth Factor Receptor
IKK	I Kappa Kinase
IL	InterLeukin
IRS	Insulin Receptor Substrate
JAK	JAnus Kinase
LOH	Loss Of Heterozygosity
LPS	LipoPolySaccharide
MAPK	Mitogen Activated Protein Kinase
MCL	Mantle Cell Lymphoma
Mdm2	Mouse double minute 2 homolog
MEK	Mitogen-activated protein kinase kinase
mLST8	Regulatory-associated protein of mTOR
MM	Multiple Myeloma
MPN	Myelo Proliferative Neoplasms
mRCC	metastatic Renal Cell Carcinoma
mSIN1	mammalian Stress-activated protein kinase Interacting protein 1
mTOR	mammalian Target Of Rapamycin
mTORC	mTOR Complex
NA	Not Available
NDRG1	N-myc Downstream-Regulated Gene 1 protein
NET	NeuroEndocrine Tumor
NF	NeuroFibromin
NF-κB	Nuclear Factor-κB
NSCLC	Non-Small Cell Lung Cancer
ODC	Ornithine DeCarboxylase
OS	Overall Survival
PBRM1	Protein PolyBroMo-1
PDAC	Pancreatic Ductal Adenoma Carcinoma
PDGF	Platelet-Derived Growth Factor
PFK	Phospho-Fructo Kinase
PFS	Progression-Free Survival
PIP	PhosphatidylInositol Phosphate
PI3K	PhosphoInositide3-Kinase
PI3KC	PhosphIinositide-3-Kinase Catalytic
PKC	Proteine-Kinase C
PNET	Pancreatic NeuroEndocrine Tumor
PP2A	Protein Phosphatase 2A
PRAS40	Proline-Rich AKT Substrate 40
Protor	Protein observed with RICTOR
PRR	Proline-Rich Protein
PTEN	Phosphatase and tensin homolog on chromosome 10
p70*^S6K1^*	p70 ribosomal protein S6 Kinase 1
p90*^RSK1^*	p90 Ribosomal S6 Kinase 1
RAF	Rapidly Accelerated Fibrosarcoma
Raptor	Regulatory-associated protein of mTOR
RCC	Renal Cell Carcinoma
REDD1	DNA Damage and Development 1
RET	REarranged during Transfection
Rictor	Rapamycin insensitive companion 44 of mTOR
RTK	Receptor Tyrosine Kinase
SGK	Serum and Glucocorticoid Kinase
SRE-BP1	Sterol Regulatory Element-Binding Protein 1
STAT	Signal Transducer and Activator of Transcription
STK	Serine Threonine Kinase
S6RP	S6 Ribosomal Protein
TBC1D7	TBC1 Domain family Member 7
Tel2	Telomere maintenance 2
TFEB	Transcription Factor EB
TKI	Tyrosine Kinase Inhibitor
TNF-α	Tumor Necrosis Factor α
TSC	Tuberous Sclerosis Complexes
Tti1	Tel2-interacting protein 1
TOS	mTORC1 Signaling motif
ULK	UNC-5 Like autophagy activatingKinase
VEGF	Vascular Endothelial Growth Factor
VEGFR	VEGF Receptor
VHL	von Hippel-Lindau
YKL	chitinase 3-like protein
4E-BP1	Elongation Initiation Factor (EIF)-4E Binding Protein 1
